# Research on the effectiveness and safety of bronchial thermoplasty in patients with chronic obstructive pulmonary disease

**DOI:** 10.1186/s40001-023-01319-9

**Published:** 2023-09-09

**Authors:** Tao Wang, Peng Fu, Fa Long, Shengming Liu, Siyu Hu, Qiongping Wang, Zhihui Huang, Liang Long, Wenting Huang, Fengbo Hu, Jingfan Gan, Hongbo Dong, Guomei Yan

**Affiliations:** 1 University of Chinese Academy of Sciences Shenzhen Hospital, No. 4253, Songbai Road, Guangming District, Shenzhen, 518106 People’s Republic of China; 2 The First Affiliated Hospital of Jinan University, Guangzhou, People’s Republic of China; 3 Department of Pulmonary and Critical Care Medicine, The First Affiliated Hospital of Jinan University, 613 W. Huangpu Avenue, Guangzhou, 510630 People’s Republic of China

**Keywords:** COPD, Bronchial thermoplasty, Effectiveness, Safety

## Abstract

**Objectives:**

To investigate the clinical efficacy and safety of bronchial thermoplasty (BT) in treating patients with chronic obstructive pulmonary disease (COPD).

**Methods:**

Clinical data of 57 COPD patients were randomized into the control (*n* = 29, conventional inhalation therapy) or intervention group (*n* = 28, conventional inhalation therapy plus BT). Primary outcomes were differences in clinical symptom changes, pulmonary function-related indicators, modified Medical Research Council (mMRC), 6-min walk test (6MWT), COPD assessment test (CAT) score and acute exacerbation incidence from baseline to an average of 3 and 12 months. Safety was assessed by adverse events.

**Results:**

FEV_1_, FEV_1_(%, predicted) and FVC in both groups improved to varying degrees post-treatment compared with those pre-treatment (*P* < 0.05). The Intervention group showed greater improving amplitudes of FEV_1_ (F_time × between groups_ = 21.713, *P* < 0.001) and FEV_1_(%, predicted) (F_time × between groups_ = 31.216, *P* < 0.001) than the control group, and there was no significant difference in FVC variation trend (F_time × between groups_ = 1.705, *P* = 0.193). mMRC, 6MWT and CAT scores of both groups post-treatment improved to varying degrees (Ps < 0.05), but the improving amplitudes of mMRC (F_time × between groups_ = 3.947, *P* = 0.025), 6MWT (F_time × between groups_ = 16.988, *P* < 0.001) and CAT score (F_time × between groups_ = 16.741, *P* < 0.001) in the intervention group were greater than the control group. According to risk assessment of COPD acute exacerbation, the proportion of high-risk COPD patients with acute exacerbation in the control and intervention groups at 1 year post-treatment (100% vs 65%, 100% vs 28.6%), inpatient proportion (100% vs 62.1%; 100% vs 28.6%), COPD acute exacerbations [3.0 (2.50, 5.0) vs 1.0 (1.0, 2.50); 3.0(3.0, 4.0) vs 0 (0, 1.0)] and hospitalizations [2.0 (2.0, 3.0) vs 1.0 (0, 2.0); 2.0 (2.0, 3.0) vs 0 (0, 1.0)] were significantly lower than those pre-treatment (*P* < 0.05). Besides, data of the intervention group were significantly lower than the control group at each timepoint after treatment (*P* < 0.05).

**Conclusions:**

Combined BT therapy is superior to conventional medical treatment in improving lung function and quality of life of COPD patients, and it also significantly reduces the COPD exacerbation risk without causing serious adverse events.

**Supplementary Information:**

The online version contains supplementary material available at 10.1186/s40001-023-01319-9.

## Background

COPD is a common lung disease worldwide [1]. Based on the latest epidemiological data, COPD is the third leading cause of disease-related death [2]. Nearly, 25% of COPD patients have a history of asthma [3], and about 50% of asthma patients develop the COPD overlap syndrome. ACO (asthma–COPD overlap) patients must rely on long-term drugs to suppress their exacerbation and acute attacks [4, 5].

It is currently believed that COPD is chronic airway limitation caused by airway remodeling and decreased lung compliance due to small airway inflammation, oxidative stress and lung parenchymal destruction [6, 7]. In addition, the pathogenesis of COPD may also be related to the imbalance of airway neuroregulation. Some research suggests that patients with respiratory system diseases such as COPD and asthma are associated with excessive activation of pulmonary neuroendocrine cells (PNECs) [8]. PNECs are distributed in small intrapulmonary bronchi, pulmonary parenchyma and alveolar junction, which can sense the change in nicotine content [9]. PNECs and their secreted CGRP and 5-HT are closely related to COPD, which may participate in pathological processes of COPD, such as airway inflammatory response and bronchial smooth muscle contraction [8, 10–12].

Based on the above discussion, the pathogenesis of COPD is quite complex, which involves several links, such as airway inflammation, immunoregulation, airway neuroregulation and airway remodeling, but the precise mechanisms remain to be fully illustrated. The existing therapeutic means and methods cannot achieve satisfactory effects, the lung function of COPD patients shows a gradually declining trend year by year, and repeated acute exacerbations may occur.

Bronchial thermoplasty (BT) uses radiofrequency catheter to release radiofrequency energy in the airway wall at a specified location, converts it into heat energy and acts on bronchial smooth muscle cells to ablate the thickened airway smooth muscle layer and reduce airway responsiveness [4]. Numerous studies have confirmed the effectiveness and safety of BT therapy for refractory severe asthma [13–15]. However, its mechanism of action remains to be further illustrated.

Recent studies have found the role of BT in airway smooth muscle and airway epithelium, which can reverse airway remodeling [16]. Moreover, apart from the influence on airway smooth muscle, BT can also decrease the number of PNECs, affect the airway autonomic regulation, and down-regulate the airway neuronal excitability and nervous reflex [17–19]. More and more studies have discovered that COPD shares certain similarities to asthma in terms of the pathogenesis: COPD and asthma share similarities such as airway remodeling during disease development [20]. Some COPD patients develop pathological airway smooth muscle hyperplasia change [21, 22]. In addition, abnormality in neuroendocrine cells and disturbance of airway neuroregulation may also be observed in COPD [8, 23]. Therefore, the mechanism of action of BT may cover the pathogenesis of COPD at the same time. In this regard, it is possible to improve airway mucus secretion and regulate airway smooth muscle through rearranging epithelial cells and affecting the airway neuroendocrine cells, thus exerting a certain therapeutic effect on COPD. Some individual cases report that BT has a good therapeutic effect on ACO patients [24]. Our previous study also discovered that BT was also effective on ACO patients, which cannot be completely explained by the role of BT in airway smooth muscle [25].

Clinically used drugs for COPD are limited by poor efficacy and adverse reactions [26], which will increase physical, psychological and economic burdens on the patients, and induce irregular drug use as well as poor treatment compliance. Consequently, it is urgently needed to search for treatments with better effects. Currently, BT has not been applied in COPD treatment. Thus, the effectiveness of BT treatment on COPD patients deserves further exploration. This study compared and observed changes in COPD symptoms including lung function, hormone dosage, wheezing, shortness of breath after exercise in COPD patients pre- and post-BT, so as to provide more evidence for related treatment.

## Methods

### Study design

This was a randomized pilot study of COPD patients at the Department of Respiratory and Critical Care Medicine, the University of Chinese Academy of Sciences Shenzhen Hospital. The study was approved by our hospital ethics committee before the enrollment of any subjects. All participants in the study provided informed consent for treatment and data collection (GKDSY-LL-XJS-2018003).

### Sample size calculation

According to previous research, we chose CAT scores as the primary outcome [27–29]. The sample size was calculated based on our previous pilot study in which CAT scores yielded an effect size of 0.869 [25]. Hence, to have a power of 80% (β = 0.20) using a two-sided α = 0.05 and a hypothetical dropout rate of 20%, a minimum of sample size should be 26 participants per group (52 in total), as determined using G*Power 3.1.9.2. In our study, there were 29 and 28 patients with COPD in the control group and the intervention group, respectively. Accordingly, the sample size of our study was appropriate.

### Randomization and blindness

An independent nurse assigned subjects in the intervention group according to a computer-generated randomization list. The nurse informed the doctor after the subjects had provided the informed consent and been included in the study. Study nurses during follow-ups were blinded to the treatment status of the patients.

### Patient groups

**Fig. 1:**
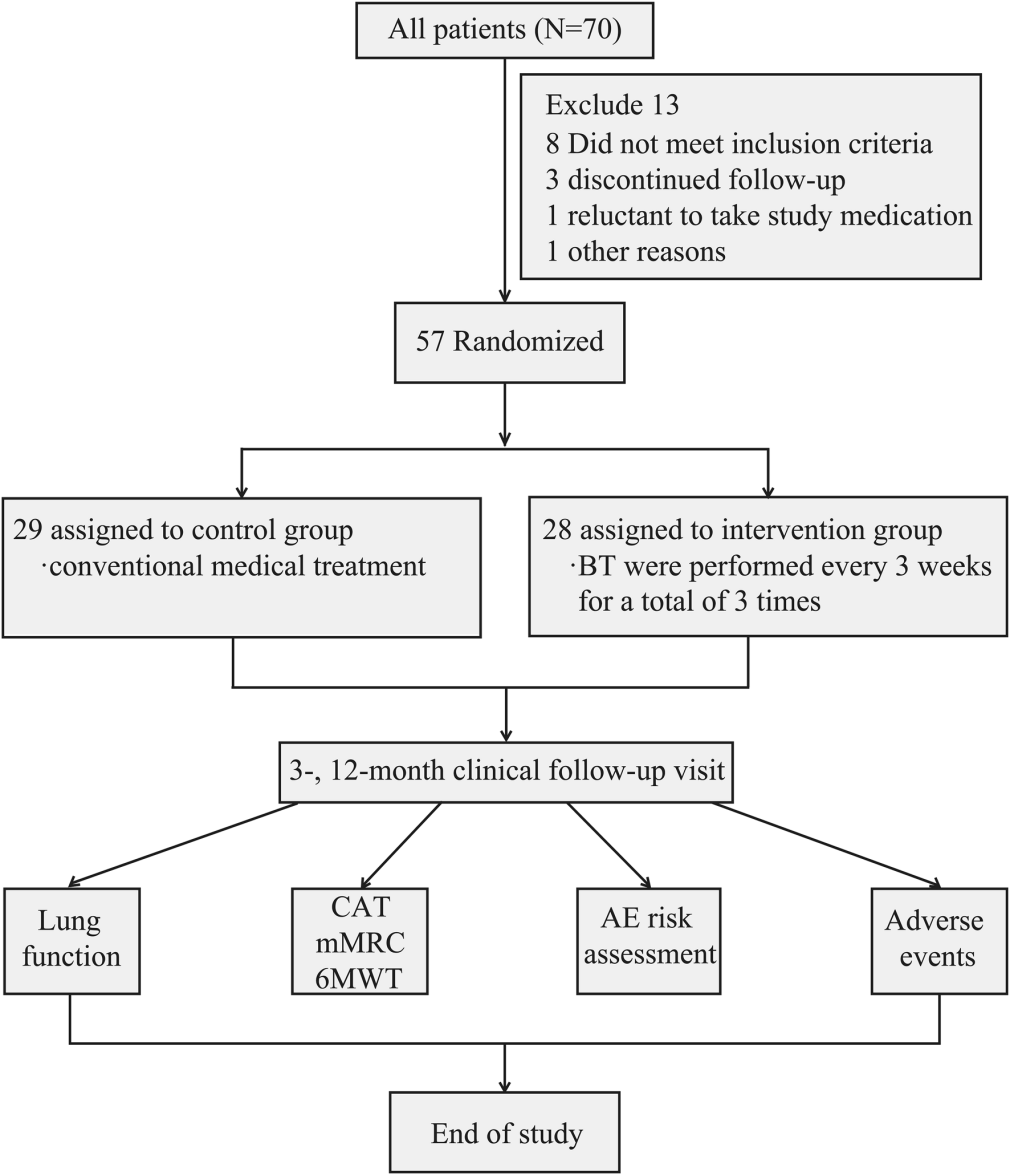
Flow diagram of study design and patient grouping

A total of 57 moderate-to-severe COPD patients were recruited consecutively between January 2019 and March 2021 at our hospital (Shenzhen Hospital of the Chinese Academy of Science), and then randomly divided into the control group and the intervention group. The patients in the control group (*n* = 29) were treated with conventional medical treatment in the stable COPD period, while those in the intervention group (*n* = 28) were treated with conventional medical treatment combined with BT ablation in the normalized stable COPD period (Fig. 1). All patients received high doses of ICS in the past year and were using LABAs and LAMAs.

### Inclusion and exclusion criteria

Inclusion criteria: (1) age between 40 and 75 years; (2) a diagnosis of COPD in accordance with the Global Initiative for Chronic Obstructive Lung Disease (GOLD) guidelines for at least 2 years [2]; (3) classification as moderate or severe (GOLD grades II–IV); (4) subjects who could provide medical records indicating the number of hospitalizations in the preceding year as a result of COPD; (5) patients who were able to document or state changes in their condition more completely; (6) subjects who understood the purpose of the trial, agreed to participate in the study and signed an informed consent form.

Patients with allergies to relevant drugs in this study, acute respiratory infection prior to admission, acute COPD exacerbation within 2 weeks, communication disorders or mental diseases, those who were unable to complete three BT sessions for various reasons, other respiratory diseases, such as asthma, cystic fibrosis, bronchiectasis, mechanical upper airway obstruction, and so on, and patients who did not consent to long-term follow-up or were diagnosed with another condition that limited life expectancy were excluded from the analysis.

A complete list of the inclusion and exclusion criteria is provided in the Additional file 1: Table S1.

### Emphysema evaluation

Emphysema was evaluated by HRCT according to the method reported previously [30, 31]. Briefly, HRCT findings were evaluated at three anatomical levels at full inspiration; near the superior margin of the aortic arch, at the level of the carina, and at the level of the orifice of the inferior pulmonary veins. The low attenuation area (LAA) was visually scored in each bilateral lung field according to the method of Goddard et al. [32]. Total scores were calculated, and the severity of emphysema was graded as follows: score 0, LAA < 5%; score 1, 5% ≤ LAA < 25%; score 2, 25% ≤ LAA < 50%; score 3, 50% ≤ LAA < 75%; and score 4, 75% ≤ LAA. Thus, the total emphysema scores ranged from 0 to 24. The severity of emphysema was graded according to the total score into mild (total score ≤ 8 points), moderate (8 points < total score ≤ 16 points) and severe (total score > 16 points).

### BT procedure

Three BT sessions were performed by the same experienced respiratory interventional physician at intervals of ≥ 3 weeks. The first session was bronchus of right lower lung lobe + right main bronchus BT. The second session was bronchus of left lower lung lobe + left main bronchus BT. The third session was bilateral upper lobe BT. BT was mainly completed by the Alair bronchial thermoforming system (Boston Scientific Corporation, USA Model: M005ATS25010). The BF260 bronchoscope was purchased from Olympus Company (Japan). Under direct view of the bronchoscope, the ablation probe was inserted into the airways one by one from small airways ≥ 3 mm up to the lobar bronchial opening from the distal to proximal end. Each radiofrequency ablation lasted for 10 s. The heating catheter was moved proximally about 5 mm for the next ablation. The ablation was completed until all bronchi 3–10 mm in diameter within the selected lung lobe were visible under the microscope. The whole process was continuous, orderly and not repetitive. Prednisone (30 mg) was administered orally once daily for 3 days preoperatively, on the operation day, and 1 day postoperatively. Meanwhile, patients took antibiotics to prevent infection. The original maintenance medication remained unchanged [33].

### Follow-up

Initially, all patients in the two groups were required to pay treatment visit at 1 month after the procedure. After the last treatment visit (designated as time 0), clinic visits were scheduled at 3 and 12 months. Subjects were contacted by telephone on days 1 and 7 after each treatment visit and monthly after the visit at month 3.

### Data collection and outcome measures

During initial clinical examinations, patient demographics were recorded for each patient,, including age, gender Body Mass Index (BMI), smoking history, smoking amount, smoking status, disease course, emphysema score, Fractional excretion of exhaled nitrogen (FeNO), blood eosinophil percentage, absolute eosinophil count, and comorbidities. Outcome measures analyzed included procedural data (including procedure time, anaesthesia type, number of activation of the BT and length of hospital stay), modified Medical Research Council (mMRC) scores, 6MWT, CAT scores, pulmonary function-related indices [FEV_1_, FEV_1_(%, predicted), FVC and FEV_1_/FVC], and the number and rate of COPD exacerbations, as well as hospitalizations resulting from such exacerbations at 3 and 12 months after procedure.

The CAT scoring questionnaire included cough, chest tightness, expectoration, sleep, mental, daily living limitations, dyspnea with increased activity, and confidence in outdoor activities. Each aspect was scored on the 0–5 scale based on severity. The total score was 40 points, with scores from 0 to 10, 11 to 20, 21 to 30 and 31 to 40 representing the “low”, “medium”, “high” and “very high” impact of the disease on a person’s health status [13, 34]. The mMRC scoring standards included [14]: 0 point: no breathlessness except on strenuous exercise; 1 point: shortness of breath when hurrying on the level or walking up a slight hill; 2 points: slower walking than people of same age on the level because of breathlessness or necessity to stop to catch breath when walking at their own pace on the level; 3 points: necessity to stop for breath after walking ~ 100 m or after few minutes on the level; 4 points: too breathless to leave the house, or breathless when dressing or undressing. Chest imaging examination: patients underwent routine chest X-ray 1 day postoperatively, and repeat chest CT scan 12 months postoperatively. An exacerbation of COPD was defined as the worsening of respiratory symptoms beyond normal day-to-day variation, often accompanied by increased local and systemic inflammation resulting from infection, pollution or other airway insults, which necessitated a change in medication [2].

### Safety

All patients underwent electrocardiogram, blood routine, urine routine, blood biochemistry, liver function, renal function and other tests before and after the study. An adverse event was recorded for any participant who required admission of longer than 48 h, or any participant who was readmitted to hospital for any cause within 30 days of any procedure.

### Statistical analysis

SPSS26.0 was employed for data recording and analysis. Normally distributed continuous data conforming to homogeneity of variance were compared by parametric test (independent samples *t* test), and expressed as mean ± standard deviation ($\overline{x}$ ± s). Abnormally distributed data were compared by Mann–Whitney U test and expressed as median [interquartile range (IQR)]. Categorical and enumeration data were presented as frequency (rate) and compared by Chi-square test or Fisher test. Through repeated measures ANOVA, indicators pre-treatment, 3 and 12 month post-treatment were compared and analyzed. Shapiro–Wilk test was used for normal distribution. Mauchly’s spherical hypothesis test was also adopted for analysis. Greenhouse and Geisser was utilized to correct the non-equal variable covariance matrices. A separate effect test was applied for significant interaction, otherwise, a main effects test was performed. Bonferroni correction was conducted to compare timepoints within groups. *P* < 0.05 (two-sided) represented statistical significance.

## Result

### Baseline demographics and clinical characteristics

Baseline information of the study population is reported in Table 1. These were two groups of patients with severe COPD, predominantly men (91.2%), with the mean FEV_1_/FVC of 48.22% and the mean FEV_1_(% predicted) of 36.08%, very symptomatic despite triple therapy and limited in their exercise capacity. Approximately 52.6% and 31.6% of the COPD patients had severe- or poorly controlled COPD at baseline, while more than 78.9% and 19.3% of the COPD patients showed a high- and very high impact of the disease on their health status(CAT) in the both groups. There was no significant difference between the two groups in age, gender, BMI, course of disease, pack-year, emphysema score, FeNO, and other general data (*P* > 0.05). Combined with BT-treated patients and controls were comparable at baseline.

**Table 1:** Baseline characteristics of the two groups of patients

Characteristic	Total (*n* = 57)	Control group (*n* = 29)	Intervention group (*n* = 28)
Age (year)	67.32 ± 8.56	66.48 ± 9.89	68.18 ± 6.99
Gender, *n* (%)			
Male	52 (91.2%)	25 (86.2%)	27 (96.4%)
Female	5 (8.8%)	4 (13.8%)	1 (3.6%)
BMI (kg/m^2^)	22.20 ± 3.80	22.08 ± 3.08	22.33 ± 4.48
Course of smoking (year)	32.96 ± 16.67	29.0 ± 18.70	37.04 ± 13.38
Current smokers, *n* (%)	22 (38.6%)	11 (37.9%)	11 (39.3%)
Pack-years	40 (20, 60)	30 (9.00, 55.00)	40 (30.00, 60.00)
More than 10 pack-year, *n* (%)	48 (84.2%)	22 (75.9%)	26 (92.9%)
Years diagnosed with COPD (year)	10 (6, 14)	10.0 (7.0, 20.0)	10.0 (6.0, 10.0)
Goddard score	9.11 ± 2.88	9.34 ± 2.61	8.86 ± 3.16
Emphysema, *n* (%)	47 (82.5%)	23 (79.3%)	24 (85.7%)
GOLD grade, *n* (%)			
II	9 (15.8%)	5 (17.2%)	4 (14.3%)
III	30 (52.6%)	15 (51.7%)	15 (53.6%)
IV	18 (31.6%)	9 (31%)	9 (32.1%)
Number of exacerbation in the past 12 months	3 (3, 4)	3 (2.5, 5)	3 (3, 4)
2 times, *n* (%)	13 (22.8%)	7 (24.1%)	6 (21.4%)
3 times, *n* (%)	18 (31.6%)	8 (27.6%)	10 (35.7%)
More than 3 times, *n* (%)	26 (45.6%)	14 (48.3%)	12 (42.9%)
Number of hospitalization for COPD exacerbation in the past 12 months	2 (2, 3)	2 (2, 3)	2 (2, 3)
Once, *n* (%)	3 (5.3%)	2 (6.9%)	1 (3.6%)
2–3 times, *n* (%)	49 (86%)	23 (79.3%)	26 (92.9%)
More than 3 times, *n* (%)	5 (8.8%)	4 (13.8%)	1 (3.6%)
Comorbidity, *n* (%)	20 (35.1%)	12 (41.4%)	8 (28.6%)
Hypertension *n* (%)	14 (24.6%)	8 (27.6%)	6 (21.4%)
Diabetes (%)	7 (12.3%)	3 (10.3%)	4 (14.3%)
Coronary heart disease (%)	7 (12.3%)	4 (13.8%)	3 (10.7%)
Inhalation therapy			
LABA/LAMA/ICS, % (*n*)	57 (100%)	29 (100%)	28 (100%)
Lung function			
FEV_1_ (L)	0.91 ± 0.41	0.93 ± 047	0.88 ± 0.34
FEV_1_ (%, predicted)	36.08 ± 12.34	37.52 ± 13.90	34.60 ± 10.54
FVC (L)	1.86 ± 0.55	1.85 ± 0.59	1.87 ± 0.51
FEV_1_/FVC (%)	48.22 ± 13.36	49.61 ± 15.68	46.77 ± 10.54
mMRC score	2.96 ± 0.68	3.03 ± 0.73	2.89 ± 0.63
6MWT (m)	248.60 ± 63.50	243.10 ± 75.29	254.29 ± 49.18
COPD Assessment Test	27.81 ± 3.9	27.93 ± 3.45	27.68 ± 4.51
Medium impact, *n* (%)	1 (1.8%)	0 (0%)	1 (3.6%)
High impact, *n* (%)	45 (78.9%)	25 (86.2%)	20 (71.4%)
Very high impact, *n* (%)	11 (19.3%)	4 (13.8%)	7 (25%)
FeNO (ppb)	31.96 ± 12.56	34.38 ± 12.54	29.46 ± 12.30
Peripheral eosinophil count (× 10^9^/L)	0.22 ± 0.18	0.27 ± 0.20	0.17 ± 0.15
Percentage of blood eosinophils (%)	2.50 (1.05, 4.55)	2.90 (1.50, 4.80)	1.90 (0.40, 4.35)

Control group (*n* = 29) received conventional medical treatment with normalized stable COPD, and observation group (*n* = 28) received conventional medical treatment plus BT ablation during the normalized stable COPD period. Data were expressed as mean ± SD or number, percentage, median (interquartile range), with significance level at *P* < 0.05

*BMI* body mass index, *FeNO* fractional excretion of exhaled nitrogen, *ICS* inhaled corticosteroid, *LABA* long-acting beta-agonist, *LAMA* long-acting muscarinic antagonist

### BT activation frequencies and endoscopic changes

**Fig. 2:**
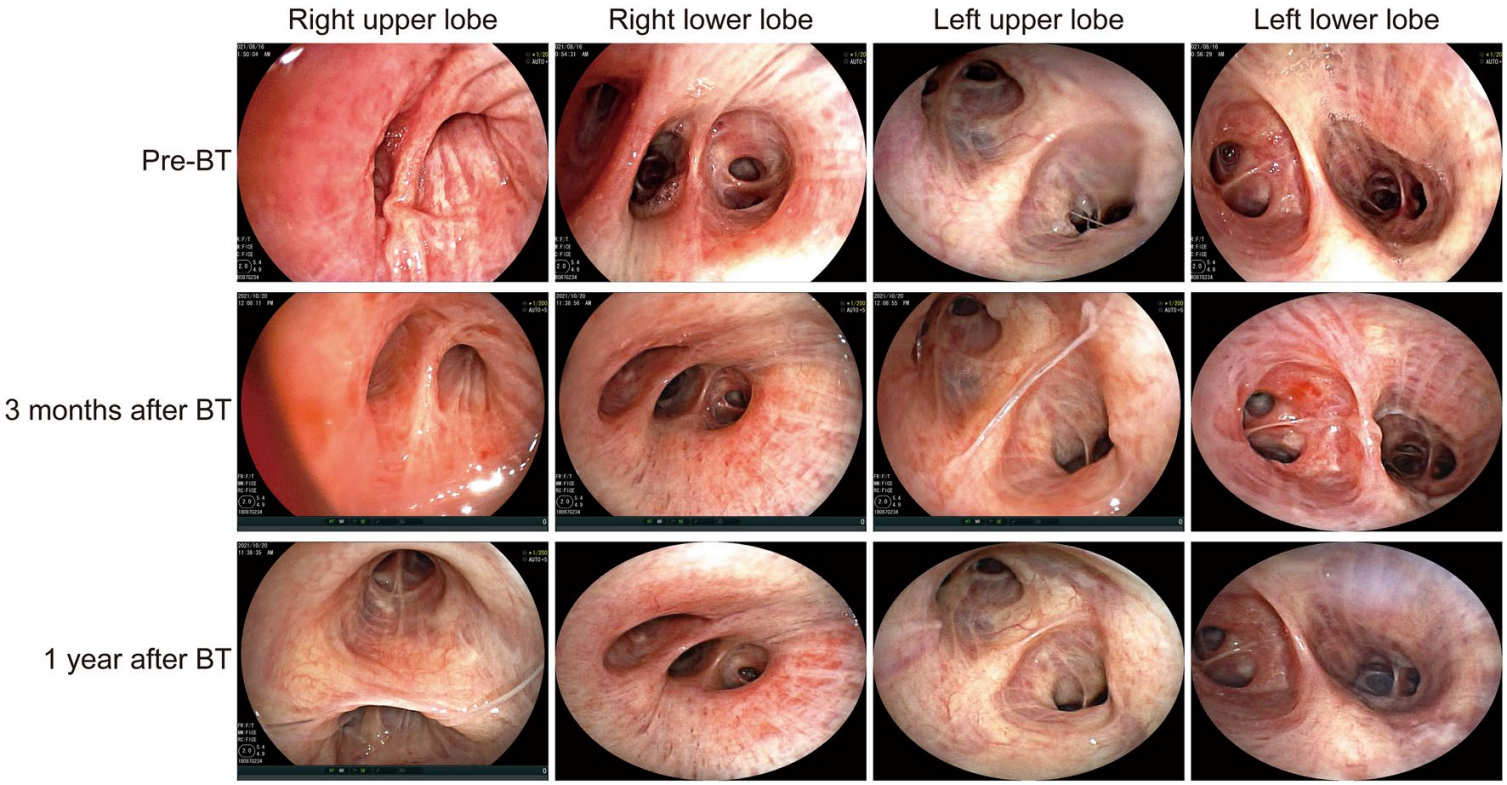
Endoscopic changes before and after BT

For the intervention group treated with combined BT therapy, the effective radiofrequency activations of right, left lower lobe, bilateral upper lung lobes, and total effective radiofrequency activations were (61.47 ± 12.59), (65.53 ± 13.91), [79.50 (63.75, 92.50)], and (207.03 ± 33.34), respectively. The intervention group after completing the above-mentioned three sessions of BT treatment was compared with pre-treatment. As a result, the airway mucus secretion in the patients decreased after each BT treatment compared with that before, accompanied by alleviated airway mucosal congestion and edema (Fig. 2).

### Comparison of lung function pre- and post-treatment between two groups

**Fig. 3:**
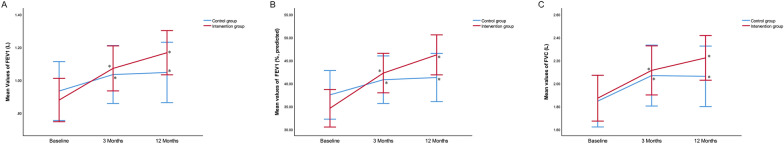
Comparison of lung function between the two groups of patients before and after treatment. **A** Change from baseline in FEV_1_ over 12 months; **B **change from baseline in FEV_1_(%, predicted); **C** change from baseline in FVC over 12 months. Mean values are shown for all subjects for whom data were available at the given timepoints, error bars are 95% CI. *P < 0.05 compared with the baseline. FEV_1_, the first–second forced expiratory volume; FVC, forced vital capacity

Through repeated measures ANOVA, changes in FEV_1_, FEV_1_(%, predicted) and FVC of different groups were judged (Table 2 and Fig. 3). There was an interaction between group and time in FEV_1_ (F = 21.713, *P* < 0.001). Changes in different groups exerted different effects on FEV_1_, with inconsistent trends in FEV_1_ elevation. Comparatively, FEV_1_ increased faster in the intervention group than the control group. Intervention and time factors were tested for separate effects. Thus, difference was not significant between the control and intervention groups pre-treatment (F = 0.525, *P* = 0.475). At each timepoint post-treatment (3 and 12 month post-treatment), FEV_1_ of the intervention group increased relative to the control group, but with no statistical significance (F = 0.039, *P* = 0.845; F = 0.825, *P* = 0.372). In intra-group comparison, differences were significant between two groups at each timepoint (pre-treatment, 3 and 12 month post-treatment, the control group: F = 24.590, *P* < 0.001; the intervention group: F = 108.528, *P* < 0.001) (Table 2 and Fig. 3A).

**Table 2:** Mean changes from baseline in key outcome measures at 3 and 12 months in two groups of patients

	Control group		Intervention group		F_time × between groups_/P value	F_main effect between groups_/P value	F_time main effect_/P value
	3 months after treatment	12 months after treatment	3 months after treatment	12 months after treatment			
FEV1	0.10 ± 0.04	0.11 ± 0.11	0.17 ± 0.06	0.24 ± 0.15	21.713/< 0.001	–	–
FEV_1_(%, predicted)	3.29 ± 1.72	3.77 ± 4.98	6.58 ± 3.02	9.19 ± 6.58	31.216/< 0.001	–	–
FVC	0.22 ± 0.40	0.22 ± 0.42	0.2 5 ± 0.19	0.34 ± 0.27	1.705/0.193	0.259/0.613	29.280/< 0.001
mMRC	− 0.41 ± 0.57	− 0.52 ± 0.63	− 0.68 ± 0.55	− 1.00 ± 0.67	3.947/0.025	–	–
6MWT	24.83 ± 8.78	31.52 ± 25.49	42.79 ± 17.53	78.07 ± 36.20	16.988/< 0.001	–	–
CAT	− 2.76 ± 1.50	3.52 ± 1.94	− 4.93 ± 2.51	− 7.36 ± 3.64	16.741/< 0.001	–	–

F_time × between groups:_ interaction effect of group and time; F_between groups:_ main effect between groups; F_time: time_ main effect. Data were expressed as mean ± standard deviation for changes from baseline to 3 and 12 months, with significance level at *P* value < 0.05

*FEV*_*1*_ forced expiratory volume in the first–second, *FVC* forced vital capacity, *mMRC* modified Medical Research Council, *6MWT* 6-min walk test, *CAT* COPD assessment test

The FEV_1_(%, predicted) showed a group–time interaction (F = 31.216, *P* < 0.001). Different groups had different effects on FEV_1_(%, predicted), with inconsistent change magnitudes between two groups. Through inter-group comparison, the intervention group had higher FEV_1_(%, predicted) than the control group at 3 and 12 month post-treatment, but with no statistical significance (F = 0.136, *P* = 0.715; F = 1.692, *P* = 0.204). According to intra-group comparison, the control (F = 13.794, *P* < 0.001) and intervention (F = 112.178, *P* < 0.001) groups showed statistical significance among pre-treatment, 3 and 12 month post-treatment (Table 2 and Fig. 3B).

There was no interaction between group and time for FVC (F = 1.705, *P* = 0.193). Therefore, intervention and time factors were tested for main effects. Consequently, the main effect on FVC was not statistically significant among different groups (F = 0.259, *P* = 0.613), while that of time factor on FVC was statistically significant (F = 29.280, *P* < 0.001). FVC was significantly different at different timepoints (pre-treatment, 3 and 12 month post-treatment) (*P* < 0.001). Therefore, FVC of the two groups post-treatment was improved compared with that before treatment (Table 2 and Fig. 3C).

### Life quality and exercise tolerance (scoring system) comparison

**Fig. 4:**
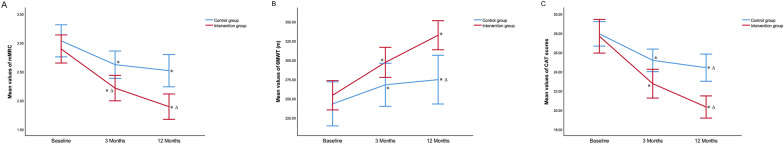
Comparison of mMRC, 6MWT and CAT scores between the two groups of patients. **A** Change from baseline in mMRC over 12 months; **B** change from baseline in 6MWT ove12 months; **C** change from baseline in CAT cores over 12 months. Mean values are shown for all subjects for whom data were available at the given timepoints, error bars are 95% CI. *P < 0.05 compared with the baseline, ^Δ^*P* < 0.05 compared with the control group (*P* < 0.05). *mMRC* modified Medical Research Council, *6MWT* 6-min walk test, *CAT* COPD assessment test

Effects of different groups on mMRC, 6MWT and CAT were judged by repeated measures ANOVA (Table 2). There was a group–time interaction for mMRC (F = 3.947, *P* = 0.025). Changes in different groups had significantly different effects on mMRC. Between-group factors and time were tested for separate effects. In inter-group comparison, difference between the control and the intervention group was not significant pre-treatment (F = 0.015, *P* = 0.523), but that was significant at 3 months (F = 5.642, *P* = 0.025) and 12 month post-treatment (F = 10.446, *P* = 0.003). Upon intra-group comparison, the control (F = 9.646, *P* = 0.001) and the intervention (F = 45.098, *P* < 0.001) groups had statistical significance at pre-treatment, 3 and 12 month post-treatment (Table 2 and Fig. 4A).

There was a group–time interaction for 6MWT (F = 16.988, *P* < 0.001). Specifically, 6MWT of different groups had different trends over time, with differences in change magnitude. Group and time factors were tested for separate effects. In between-group comparison, the intervention group was higher than the control group at pre-treatment, 3 and 12 month post-treatment. Noteworthily, difference was not significant at pre-treatment (F = 0.306, *P* = 0.585) and 3 month post-treatment (F = 2.441, *P* = 0.130), but significant at 12 month post-treatment (F = 9.205, *P* = 0.005). In intra-group comparison, the control (F = 112.042, *P* < 0.001) and intervention (F = 84.121, *P* < 0.001) group showed significant changes among pre-treatment, 3 and 12 month post-treatment (Table 2 and Fig. 4B).

There was an interactive effect between group and time for CAT (F = 16.741, *P* < 0.001). CAT of different groups had different trends over time, with different change magnitudes. Between-group factors and time were tested for separate effects. In between-group comparison, the intervention group was lower than the control group at pre-treatment, 3 and 12 month post-treatment. Difference between two groups at pre-treatment was not significant (F = 0.013, *P* = 0.911), but that was significant at 3 months (F = 5.880, *P* = 0.022) and 12 month post-treatment (F = 21.104, *P* < 0.001). Intra-group comparison revealed significant changes in CAT scores between the control (F = 57.986, *P* < 0.001) and the intervention (F = 84.636, *P* < 0.001) groups at pre-treatment, 3 and 12 month post-treatment. In addition, CAT gradually decreased with the passage of time (Table 2 and Fig. 4C).

### Acute exacerbation risk of two groups of patients before and after treatment

**Fig. 5:**
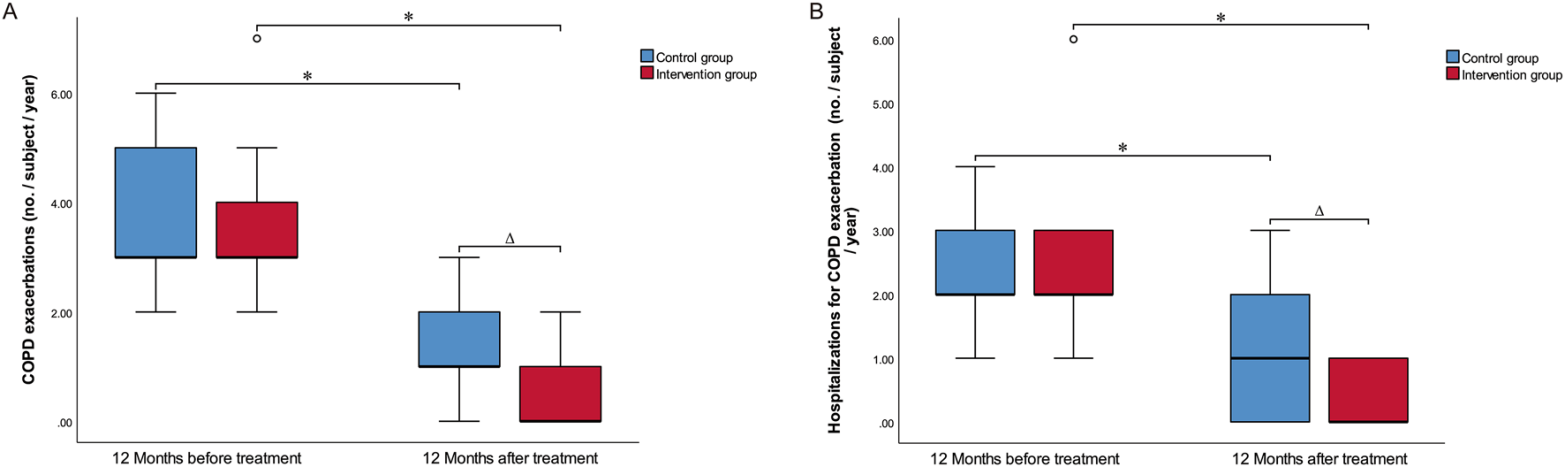
Comparison of exacerbations for COPD between the two groups of patients before and after treatment. **A** Total exacerbations and **B** hospitalisations for COPD at baseline and 12 months after treatment. Mean values are shown for all subjects for whom data were available at the given timepoints, error bars are 95% CI. *P < 0.05 compared with the 12 months before treatment. ^Δ^*P* < 0.05 compared with the control group (*P* < 0.05). *BT* bronchial thermoplasty

The COPD acute exacerbation times and hospitalizations due to COPD acute exacerbations between two groups were compared. In in-group comparison, the total number of COPD acute exacerbations and hospitalizations due to COPD acute exacerbations in both groups at 1 year after treatment apparently decreased compared with those before treatment (*P* < 0.05). While in inter-group comparison, the total number of COPD acute exacerbations and hospitalizations due to COPD acute exacerbations in intervention group significantly decreased relative to those in the control group at 1 year after treatment (*P* < 0.05, Fig. 5).

Assessment of COPD acute exacerbation risk: based on the number of acute exacerbations in the previous year, it is assessed as a high-risk group for exacerbations if there are 2 or more moderate/severe exacerbations in a year, or one or more hospitalizations for an exacerbation.

In the comparison of the proportion of high-risk patients with COPD acute exacerbation, the within-group comparison showed that the proportion of high-risk patients after treatment in that the control group decreased to 65.5%, and that in the intervention group reduced to 28.6%. Using paired Chi-square test, the proportion of high-risk patients with acute exacerbation in the control group and intervention group was statistically significant before and after treatment. The comparison between groups showed that the proportion of high-risk patients with acute exacerbation in the intervention group after treatment was significantly lower than that in the control group (X^2^ = 7.800, *P* = 0.005), as shown in Table 3.

**Table 3:** Assessment of COPD acute exacerbation risk between the two groups of patients

Project	Group	Time		Z/X^2^	*P*
		1 year before treatment	1 year after treatment		
Proportion of high-risk patients with acute exacerbation of COPD (%)	Control group	29 (100%)	19 (65.5%)	8.100	0.002
	Intervention group	28 (100%)	8 (28.6%)	18.050	< 0.001
	X^2^	–	7.800		
	*P*	–	0.005		
Cumulative number of patients with acute exacerbation of COPD	Control group	3.0 (2.50, 5.0)	1.0 (1.0, 2.50)	− 4.795	< 0.001
	Intervention group	3.0 (3.0, 4.0)	0 (0, 1.0)	− 4.697	< 0.001
	*Z*	− 0.363	− 3.624		
	*P*	0.717	< 0.001		
Number of inpatients with acute exacerbation of COPD (%)	Control group	29 (100%)	18 (62.1%)	9.091	0.001
	Intervention group	28 (100%)	8 (28.6%)	18.050	< 0.001
	X^2^	–	6.443		
	*P*	–	0.011		
Cumulative number of inpatients with acute exacerbation of COPD	Control group	2.0 (2.0, 3.0)	1.0 (0, 2.0)	− 4.381	< 0.001
	Intervention group	2.0 (2.0, 3.0)	0 (0, 1.0)	− 4.960	< 0.001
	*Z*	0.009	− 3.050		
	*P*	0.993	0.002		

Data were expressed as percentage or median (interquartile range), with significance level at *P* value < 0.05

In the comparison of cumulative number of patients with COPD acute exacerbation, within-group comparison shows that the cumulative number of cases with acute exacerbation in that control group and intervention group were significantly lower after treatment (*P* < 0.001). According to the comparison between groups, the cumulative number of cases with acute exacerbation in the intervention group was significantly lower than that in the control group after treatment (Z = − 3.624, *P* < 0.001), as shown in Table 3.

In the comparison of the proportion of hospitalized patients between the two groups of patients with COPD acute exacerbations, the intra-group comparison results showed that the proportion of hospitalized patients after treatment in the control group decreased to 62.1%, while the proportion of patients treated in the intervention group decreased to 28.6%. With the paired Chi-square test, the difference in the proportion of hospitalized patients between the control group and the intervention group was statistically significant before and after treatment. The comparison between groups showed that the proportion of hospitalized patients in the intervention group after treatment was significantly lower than that in the control group (X^2^ = 6.443, *P* = 0.011), as shown in Table 3.

In the comparison of the cumulative number of inpatients with COPD acute exacerbations between the two groups, the intra-group comparison results showed that the cumulative number of cases with acute exacerbation in the control group and intervention group were significantly decreased after treatment (*P* < 0.001). According to the comparison between groups, the cumulative number of cases with acute exacerbation in the intervention group was significantly lower than that in the control group after treatment (*Z* = − 3.050, *P* = 0.002), as shown in Table 3.

### Safety analysis and comparison of respiratory adverse events in two groups of patients within 4 weeks after treatment

In the combined BT treatment group, the main adverse events in patients with COPD within 3 weeks after treatment were cough, phlegm, and short-lived wheezing. Specifically, there were cough (39 cases), increased expectoration (32 cases), short-term wheezing (32 cases), blood in sputum (8 cases), chest tightness and pain (4 cases), pneumonia (3 cases) cases), focal atelectasis (13 cases), hypoxemia (1 case), and hypercapnia (2 cases). Most adverse events resolved spontaneously 1 week after operation or disappeared after symptomatic treatment, such as sputum suction under bronchoscopy and non-invasive ventilator-assisted ventilation. In addition, no structural changes such as bronchial stenosis or tracheal dilatation or segmental atelectasis occurred during postoperative chest computed tomography (CT) follow-up. There were no patients in each group who terminated the study early due to adverse reactions or experienced serious adverse reactions within 3 weeks.

## Discussion

COPD is a chronic airway inflammatory disease characterized by incompletely reversible airflow limitation, whose symptoms include small airway mucus formation, airway wall fibrosis, and emphysema [35]. Like asthma, airway remodeling is also a major pathogenesis of COPD [36, 37]. COPD induces airway remodeling changes, such as airway wall thickening and airway smooth muscle hyperplasia. COPD-induced airway smooth muscle proliferation mainly occurs in small airways [38, 39]. The increased ASM mass caused by hyperplasia and hypertrophy significantly affects overall airway remodeling in COPD patients, which is closely related to disease severity [40, 41]. The lungs have numerous vagal parasympathetic nerves interacting with cholinergic receptors in the bronchial tree by releasing acetylcholine. Therefore, smooth muscle contraction, airway mucus secretion, and local inflammatory responses occur [42]. In COPD patients, enhanced pulmonary vagal parasympathetic nerve activity is an important contributor to the reversible distal airway obstruction [43]. These pathological changes are closely related to declined lung function and life quality in COPD patients [44–47].

Bronchodilator agents are the cornerstone of COPD treatment, but they cannot completely reverse airway remodeling. As a new interventional technique, BT is effective on improving airway remodeling and lung function and relieving COPD [48–51]. The AIR, AIR2, and RISA trials are conducted on patients with moderate-to-severe, severe and refractory severe asthma, confirming that BT improves life quality and clinical symptoms of moderate-to-severe and above asthma patients [52–54]. Asthma and COPD are chronic airway inflammatory diseases characterized by airflow limitation, which share multiple similarities, including ① common molecular targets and inflammatory mediators, ② interchanged inflammatory features during acute exacerbations and infections, and ③ changes in airway smooth muscle hyperplasia and airway remodeling [55]. However, the mechanisms of action of BT remain largely unclear, and they may be other mechanisms besides airway smooth muscle ablation [56]. BT has different effects on various airway cellular components (inflammatory cells and epithelial cells) and the complex airway wall structure to exert therapeutic effects. Since 2015, targeted lung denervation (TLD) has safely achieved long-term targeted, minimally invasive regulation of lung vagal sympathetic nerve, which has gradually developed into an emerging technology for treating COPD. BT reduces nerves in small airways and denervate the lungs, showing a therapeutic effect on COPD [18]. In individual reports, BT successfully improves lung function and clinical symptoms in childhood asthmatic COPD patients [24]. Our previous results showed that BT significantly improves lung function and life quality in ACO patients. Therefore, BT treatment combined with conventional medical treatment may better treat COPD.

This study compared the difference in the efficacy between combined BT treatment and conventional medical treatment. As a result, both treatments improved FVC, FEV_1_ and FEV_1_%predicted in a time-dependent manner, with better effect being achieved in a longer time. Therefore, both treatment modalities were effective on improving lung function. In further intergroup comparison, intervention group after combined BT treatment had higher FVC at 3 months and 1 year postoperatively than the control group, but with no significant difference. FVC is a measure of lung volume. Existing inhalation drugs primarily work by relaxing bronchial smooth muscle, dilating bronchi, and relieving airflow limitation. Combined BT treatment mainly works by ablating bronchial smooth muscle, which has only a slow and limited effect on improving lung volume in emphysema patients. FVC showed a clear upward trend. Therefore, the insignificant difference between intervention and control groups at 3 months and 1 year postoperatively is related to the short observation time. Based on relevant foreign studies, BT treatment has long-term effectiveness [13, 57, 58]. Thus, prolonging the observation time further reveals BT’s therapeutic efficacy, and combined BT treatment may achieve significantly better effect than conventional medical treatment.

Moreover, FEV_1_ and FEV_1_(%, predicted) showed group–time interactions. The trend and magnitude of FEV_1_ improvement over time were different between two group. Combined BT treatment improved more FEV_1_ and FEV_1_(%, predicted) in COPD patients. In inter-group comparison, intervention group had significantly higher FEV_1_ at 3 months and 1 year post-treatment than the control group, indicating that combined BT treatment had a better effect on improving FEV_1_, possibly because that BT primarily ablated airway smooth muscle. Airflow restriction is improved by further airway opening. As an indicator of reaction gas flow rate, BT more significantly improved FEV_1_ than conventional medical treatment, which was evident 3 months and 1 year post-treatment. However, difference in FEV_1_(%, predicted) was not significant between two groups at 3 months and 1 year post-treatment, possibly because that FEV_1_(%, predicted) was affected by patient's lung volume. There was no group–time interaction between FEV_1_/FVC, or statistical significance between-group main effect and time main effect, probably because of insignificant improvements in FEV_1_ and FVC, causing the insignificant improvement in ratio.

Lung functional changes do not accurately reflect the life quality of patients. Poor lung function does not necessarily influence on patient life quality. Conversely, some patients with good lung function develop severe clinical symptoms that significantly affect their life quality. Assessing patient life quality and exercise tolerance is important to determine the effectiveness of combined BT treatment for COPD.

The 6-min walk test is closely related to lung function parameters in COPD patients, with longer 6-min walking distance indicating better pulmonary ventilation function [59]. Intervention group had significantly higher 6-min walking distance at 3 months and 1 year post-treatment than the control group. Combined BT treatment led to greater improvements. Therefore, combined BT treatment better improves exercise tolerance in COPD patients, possibly by improving lung function.

mMRC assesses dyspnea level based on the shortness of breath symptom at the corresponding exercise intensity. Combined BT treatment significantly reduced mMRC and improved dyspnea. Considering the relatively one-sided assessment of mMRC, CAT assessment was conducted. CAT is a questionnaire based on SGRQ, reflecting life quality in COPD patients. Compared with assessing COPD life quality based on FEV_1_ alone, CAT accurately represents the current true level, which is no less than other complex health questionnaires. Similar results to mMRC were obtained. Compared with control group, combined BT treatment further reduced CAT score in COPD patients, suggesting its effect on better improving patient life quality. Furthermore, this effect was markedly effective at 3 months and 1 year post-treatment.

COPD acute exacerbation indicates an acute exacerbation of a patient’s respiratory symptoms and the need for additional treatment. After an exacerbation, about 20% of COPD patients do not return to their previous state. Declined lung function induces persistently severe COPD and reduces the life quality [60]. Moreover, COPD acute exacerbations lead to airway inflammation deterioration and hematological immune responses, significantly increasing the death risk in patients [61]. Thus, exacerbations play an important role in managing COPD disease. In this study, combined BT treatment remarkably reduced COPD acute exacerbations, and the resultant hospitalizations. Thereby, the numbers of high-risk patients and hospitalized patients decrease, suggesting that combined BT treatment partially reduces acute exacerbations in COPD patients. The lung function in COPD patients is a factor affecting their acute exacerbations. FEV_1_ is often used to predict COPD severity, acute exacerbation risk and mortality. Combined BT treatment better improved FEV_1_, reducing the COPD acute exacerbation risk.

Using long-acting anticholinergic drugs (LAMAs) is found to effectively improve lung function and reduce acute exacerbations in asthma and COPD patients [62, 63]. TLD treatment significantly reduces the exacerbation-induced hospitalizations in moderate-to-severe COPD patients [64]. BT treatment can achieve denervation, reducing the COPD acute exacerbation risk.

COPD is a chronic respiratory tract inflammatory disease. Studies have found that persistent and low-grade systemic inflammation is a factor for frequency-sensitive exacerbations [65–67]. BT acts on airway smooth muscle and airway epithelium cells, while the former cells secrete pro-inflammatory factors and cytokines [16, 68]. Therefore, BT may affect airway inflammation by ablating airway smooth muscle. BT treatment reduces airway TGF-beta, and blood eosinophil levels [69, 70]. Consequently, BT’s effect on reducing COPD acute exacerbations may associate with its effect on airway inflammation.

For safety, respiratory adverse events in both groups mostly included cough, phlegm, and short-term wheezing, which were effectively controlled in a short time. No serious adverse events such as malignant arrhythmia or death were observed in both groups within 1 year post-treatment. Therefore, BT treatment is safe and feasible for COPD patients.

Combined BT treatment better improve lung function of COPD patients than conventional medical treatment, significantly improve life quality and reduce COPD acute exacerbation. BT-related adverse reactions are controllable in the short term, without serious adverse events. However, there are some limitations of this study. First, this study did not set the sham-operation group, which might have a certain effect on the results. Therefore, clinical studies with more perfect clinical design should be conducted. Second, this work enrolled patients with moderate to severe COPD, and the severity of emphysema also varied, which might lead to the difference in BT therapeutic effect. In addition, no subgroup analysis was conducted in this work, making it impossible to further explore which type of COPD patients could gain more benefits. In the future research, patients should be further classified according to the indicators, such as the severity of emphysema, so as to explore more COPD subtypes that can benefit from BT treatment and to improve the effect of BT on treating COPD. Third, this study did not collect the airway biopsy samples of patients, as a result, the airway pathological changes before and after BT could not be explored, and the mechanism of action of BT in COPD could not be analyzed. Finally, this was a single-center randomized controlled trial with a small sample size. Because of the limited sample size of participants diagnosed with COPD in this study, the current results should be further validated by subsequent studies.

## Conclusion

Combined BT treatment can better improve lung function in COPD patients, definitely improve the patient life quality, and greatly prevent COPD acute exacerbation. Therefore, BT is a new interventional therapy for COPD patients with repeated acute exacerbations or those who fail the conventional medical treatment.

### Supplementary Information

**Additional file 1: Table S1.** Inclusion and exclusion criteria.

## Data Availability

Please contact the primary author for data requests.

## Abbreviations

BT: Bronchial thermoplasty
COPD: Chronic obstructive pulmonary disease
FVC: Forced vital capacity
FEV_1_: Forced expiratory volume in the first–second
FEV_1_/FVC: Ratio of FEV_1_/FVC
CAT: COPD assessment test
mMRC: Modified Medical Research Council
ACO: Asthma–COPD overlap
BMI: Body Mass Index
ALT: Alanine aminotransferase
AST: Aspartate aminotransferase
Cr: Creatinine
ULN: Upper limit of normal
6MWT: 6-Min walk test
LAMA: Long-acting muscarinic antagonist
LABA: Long-acting beta2-agonist
CT: Computed tomography
TLD: Targeted lung denervation
