# Supplementary material for: Research on the effectiveness and safety of bronchial thermoplasty in patients with chronic obstructive pulmonary disease
Source: Eur J Med Res. 2023 Sep 9;28:331. doi: 10.1186/s40001-023-01319-9 (PMC10492361; doi:10.1186/s40001-023-01319-9)
Supplement: Supplementary file 1 — Additional file 1: Table S1. Inclusion and exclusion criteria. [file 40001_2023_1319_MOESM1_ESM.docx]

**Table S1 Inclusion and exclusion criteria**

| Inclusion criteria | ① Aged 40-75 yearsd  ② Following diagnostic criteria of COPD in 2019 Global Initiative for COPD (GOLD Guidelines): FEV1/FVC<0.70 after bronchodilator use, 2-3GOLD level, poor conventional medical treatment effect, and repeated acute aggravated.  ③ Following definition of COPD stable stage in 2019 Global Initiative for COPD (GOLD Guidelines): stable disease, no cough, sputum, shortness of breath or other aggravating symptoms, no need to change treatment plan [2]  ④ Regular inhalant use, following recommendations for COPD patients in Group D in 2019 Global Initiative for COPD (GOLD Guidelines) [2].  ⑤ COPD patients were diagnosed as stable group D. The airflow limitation severity ranges from level II (moderate) to level IV (very severe).  ⑥ SpO_2_ ≥89% on room air during screening.  ⑦ CAT score ≥10 during screening.  ⑧ Documented history of ≥2 moderate COPD exacerbations or ≥ 1 severe COPD exacerbation causing hospitalization within 12 months before consent.  ⑨ Documented history of taking at least Long-Acting Muscarinic Antagonist (LAMA) and a Long-Acting Beta2 -Agonist (LABA) as regular respiratory maintenance medication for ≥12 months at the time of consent.  ⑩ Candidate for bronchoscopy decided by the physician or per hospital guidelines. Suitability of bronchoscopy candidates includes but is not limited to: cardiovascular fitness, ability for intubation, ability for oxygenation, no previously diagnosed high-grade tracheal obstruction, no uncorrectable coagulopathy (i.e. participant cannot stop taking blood thinning medication, except aspirin, 7 days before and not restart until 7 days after study). |
| --- | --- |
| Exclusion criteria | ① Implanted cardiac pacemakers;  ② Acute myocardial infarction within 6 weeks;  ③ Severe cardiopulmonary disease preventing bronchoscopy;  ④ Other diseases presenting with incomplete airflow limitation were excluded;  ⑤ Acute COPD exacerbation within 2 weeks;  ⑥ Allergy to anesthetics and unsuitability for bronchoscopy  ⑦ Inability to stop taking anticoagulant or antiplatelet drugs or uncorrectable coagulation disorders;  ⑧ ALT and AST ≥1.5-fold of upper limit of normal (ULN), and Cr > ULN.  ⑨ Neurological and psychiatric diseases;  ⑩ Pregnant or breastfeeding women or those planning to become pregnant in the near future;  ⑪ Serious primary diseases of heart, liver, kidney, hematopoietic system and other important organs and systems;  ⑫ Infections (including respiratory and extra-respiratory) within 2 weeks.  ⑬ Pulmonary nodule suspected of malignancy.  ⑭ Malignancy treated with radiation or chemotherapy within 2 years of consent.  ⑮ Daily use of >10 mg prednisone or its equivalent at the time of consent.  ⑯ Any disease or condition possibly interfering with study or procedure completion (e.g., severe pulmonary hypertension, emphysema, bullae, bronchial asthma, cystic fibrosis, bronchiectasis, mechanical upper airway obstruction, structural esophageal disorder, life expectancy <3 years).  Known contraindication or allergy to medications required for bronchoscopy or general anesthesia uncontrollable medically. |
| Exit criteria | ① Patients asking to withdraw after enrollment, having poor compliance, or not completing corresponding examinations as required;  ② Those not completing 3 BT procedures for various reasons;  ③ Those without follow-up for half a year;  ④ Those not evaluating the BT efficacy due to incomplete clinical data;  ⑤ Those using other treatments or drugs prohibited from concomitant use and affected judgment of effectiveness and safety. |
